# TB management in the European Union/European Economic Area: a multi-centre survey

**DOI:** 10.5588/ijtld.20.0849

**Published:** 2021-02-01

**Authors:** G. Sotgiu, S. Rosales-Klintz, R. Centis, L. D’Ambrosio, R. Verduin, A. M. Correia, A. Cirule, R. Duarte, B. Gadzheva, G. Gualano, H. Kunst, F. Palmieri, V. Riekstina, D. Stefanova, S. Tiberi, M. J. van der Werf, G. B. Migliori

**Affiliations:** 1 Clinical Epidemiology and Medical Statistics Unit, Department of Medical, Surgical and Experimental Sciences, University of Sassari, Sassari, Italy; 2 European Centre for Disease Prevention and Control, Stockholm, Sweden; 3 Servizio di Epidemiologia Clinica delle Malattie Respiratorie, Istituti Clinici Scientifici Maugeri, Istituto di Ricovero e Cura a Carattere Scientifico (IRCCS), Tradate, Italy; 4 Public Health Consulting Group, Lugano, Switzerland; 5 Verduin Public Health Consult, Oegstgeest, the Netherlands; 6 Regional Health Administration of the North, Department of Public Health, Porto, Portugal; 7 Centre of TB and Lung Diseases, Riga East University Hospital, Riga, Latvia; 8 National Reference Centre for MDR-TB, Hospital Centre Vila Nova de Gaia, Department of Pneumology; Public Health Science and Medical Education Department, Faculty of Medicine, University of Porto, Porto, Portugal; 9 The Global Fund to fight AIDS, Tuberculosis and Malaria (GFATM) Programme, Department of Management of Specialized Donor-Funded Programmes, Ministry of Health, Sofia, Bulgaria; 10 Respiratory Infectious Diseases Unit, L Spallanzani National Institute for Infectious Diseases, IRCCS, Rome, Italy; 11 Blizard Institute, Barts and The London School of Medicine and Dentistry, Queen Mary University, London, UK; 12 St Sofia University Hospital for Active Treatment of Respiratory Diseases, Sofia, Bulgaria; 13 Division of Infection, Royal London Hospital, Barts Health NHS Trust, London, UK

**Keywords:** multidrug-resistant TB, extensively drug-resistant TB, TB-HIV co-infection, infection control, workplace safety

## Abstract

**BACKGROUND:**

Essential TB care in the European Union/European Economic Area (EU/EEA) comprises 21 standards for the diagnosis, treatment and prevention of TB that constitute the European Union Standards for Tuberculosis Care (ESTC).

**METHODS:**

In 2017, we conducted an audit on TB management and infection control measures against the ESTC standards. TB reference centres in five EU/EEA countries were purposely selected to represent the heterogeneous European TB burden and examine geographic variability.

**RESULTS:**

Data from 122 patients, diagnosed between 2012 and 2015 with multidrug-resistant TB (*n =* 49), extensively drug-resistant TB (XDR-TB) (*n* = 11), pre-XDR-TB (*n* = 29) and drug-susceptible TB (*n* = 33), showed that TB diagnosis and treatment practices were in general in agreement with the ESTC.

**CONCLUSION:**

Overall, TB management and infection control practices were in agreement with the ESTC in the selected EU/EEA reference centres. Areas for improvement include strengthening of integrated care services and further implementation of patient-centred approaches.

## Abstract (FR)

**CONTEXTE:**

Les normes de soins de la TB de l’Union Européenne (ESTC) résument le niveau essentiel des soins de la TB dans l’Union Européenne/la Zone Economique Européenne (EU/EEA) en 21 normes de diagnostic, de traitement et de prévention de la TB.

**MÉTHODES:**

En 2017, nous avons réalisé un audit de la prise en charge de la TB et des mesures de lutte contre l’infection par rapport aux normes de l’ESTC. Les centres de référence TB de cinq pays d’EU/EEA ontété sélectionnés pour représenter le poids hétérogène de la TB en Europe et la variabilité géographique.

**RÉSULTATS:**

Les données de 122 patients, ayant eu, entre 2012 et 2015, un diagnostic de TB multirésistante (*n*= 49), extrêmement résistante (XDR-TB) (*n*= 11), TB pré-XDR (*n* = 29) et pharmacosensible (*n* = 33) ont montré que les pratiques du diagnostic et du traitement de la TB ont généralementété en accord avec l’ESTC.

**CONCLUSION:**

Dans l’ensemble, la prise en charge de la TB et les mesures de lutte contre l’infection ontétéen accord avec l’ESTC dans les centres de référence sélectionnés de l’EU/EEA. Les domaines restantà améliorer sont le renforcement des services de prise en charge intégrée et la mise en œuvre accrue d’approches centrées sur le patient.

## Abstract (ES)

**MARCO DE REFERENCIA:**

Los estándares europeos para el tratamiento de la TB (ESTC) resumen la atención básica de la TB en la Unión Europea y el Espacio Económico Europeo (UE/EEE) en 21 normas para el diagnóstico, el tratamiento y la prevención de la TB.

**MÉTODO:**

En el 2017, se llevó a cabo un examen del tratamiento de la TB y las medidas de control de infecciones con respecto a los ESTC. Se escogieron de manera deliberada centros de referencia de TB en cinco países de UE/EEE que representaran la heterogeneidad de la carga de morbilidad por TB y la variabilidad geográfica.

**RESULTADOS:**

Los datos de 122 pacientes diagnosticados entre el 2012 y el 2015 con TB multirresistente (*n* = 49), TB ultrarresistente (XDRTB) (*n* = 11), TB pre-XDR (*n* = 29) y TB sensible (*n* = 33) pusieron en evidencia que, en general, las prácticas de diagnóstico y tratamiento de la TB eran conformes con los ESTC.

**CONCLUSIÓN:**

En general, las prácticas de tratamiento de la TB y de control de infecciones en los centros de referencia escogidos de la UE/EEE están en consonancia con los ESTC. Las esferas que pueden mejorarse son el fortalecimiento de los servicios integrados de atención y una mayor aplicación de los enfoques centrados en el paciente.

TB REMAINS A MAJOR CLINICAL and public health threat worldwide. In 2016, 58 994 TB cases were notified in the European Union/European Economic Area (EU/EEA), with ~4% diagnosed as multidrug-resistant TB^1^ (MDR-TB, i.e., *Mycobacterium tuberculosis* resistant to at least isoniazid and rifampicin^2^). Concerns about the clinical management of TB in the EU/EEA have been raised. In 2010, a survey on TB management showed shortcomings in comparison with international standards of care, especially in patients with MDR/XDR-TB (extensively drug-resistant TB; i.e., MDR-TB with additional resistance to any fluoroquinolone and at least one of three injectable second-line drugs).^3^ The results of the survey informed the development of the European Union Standards for TB Care (ESTC).^4^

Since the ESTC publication in 2012, TB care, prevention and infection control has further developed. The End TB Strategy has defined global efforts to eliminate TB (i.e., TB incidence rate <10 per 100 000 population) by 2035;^5^ new rapid genetic testing is readily available to confirm TB and MDRTB, new anti-TB drugs and treatment regimens are being used and new patient-centred approaches have been identified to support TB patients.^6^

The aim of the present study was to ascertain whether the management of TB (both drug-susceptible and MDR/XDR-TB) in selected EU/EEA settings was consistent with standards for TB care, in particular, with the standards in the first edition of the ESTC.

## METHODS

### Study setting

A multi-centre survey was performed between July and September 2017 in five TB reference centres located in EU/EEA countries with different TB epidemiological profiles: Centre 1 (southern Europe; annual TB incidence <10/100 000; MDR-TB prevalence <5%); Centre 2 (northern Europe; annual TB incidence <20/100 000; MDR-TB prevalence <5%; Centre 3 (southern Europe; annual TB incidence >20/100 000, MDR-TB prevalence <5%); Centre 4 (central Europe; annual TB incidence >20/100 000; MDR-TB prevalence <10%); Centre 5 (eastern Europe [former Soviet Union]; annual TB incidence >20/100 000; MDR-TB prevalence >10%).

### Study population

Medical records of confirmed TB cases with MDR-, pre-XDR-, XDR- or drug-susceptible TB, diagnosed between 1 January 2012 and 31 December 2015 and with definitive treatment outcomes were considered eligible. Medical records of paediatric TB cases (≤15 years of age) and confirmed TB cases with any resistance (i.e., mono-resistance or poly-drug resistance that is not MDR- or XDR-TB) were excluded. A maximum of 40 medical records per centre were reviewed. A 3:1 ratio of MDR/XDR-TB and fully drug-susceptible TB cases (per centre) was predetermined. Consecutive cases complying with the inclusion criteria, starting from those most recently diagnosed, were selected.

### Data collection instruments

Two MS Excel-based forms (MicroSoft, Redmond, WA, USA) on TB case management and availability of drugs, used for the survey in 2010, were revised and updated.^7^ The revision process included a review of relevant international guidelines and policy documents,^8^–^17^ and a consultation with a task force convened by the European Respiratory Society to support the ESTC update.^18^,^19^

New items on diagnosis (e.g., adoption of rapid molecular tests); treatment (e.g., administration of delamanid and bedaquiline, implementation of therapeutic drug monitoring) and social protection (e.g., use of enablers and material incentives; community-based support) were included.

The data collection form on TB case management comprised three sections: 1) characteristics of participating centres; 2) patient-level data on TB prevention, diagnosis, treatment and social protection measures; and 3) assessment of key case management decisions against the ESTC. The data collection form on drug availability included three sections: 1) prescribed treatments and drugs inventory (as reported in mid-2017); 2) financing and procurement procedures; and 3) national policies and treatment guidelines (see Supplementary Tables S1–S4).

### Data collection

Data were collected by local collaborators and external auditors. Local collaborators retrieved the information and filled the Excel databases. Two external auditors verified the initial data entry and assessed compliance with the ESTC. The audit team inspected each facility to assess the infrastructure, clinical and diagnostic services, patient flow and infection control measures.

### Data analysis

Absolute and relative frequencies (percentages) were used to describe categorical variables. Means (standard deviations) or medians (interquartile ranges) were used to describe continuous numerical variables, based on their parametric distribution. The collated data were reviewed against the ESTC standards on TB diagnosis and treatment (Standards 4, 8, 10–13); TB-HIV co-infection and other comorbidities (Standards 14, 15 and 17); and public health and TB prevention (Standards 18, 20 and 21).^4^ Compliance with the ESTC was expressed as a percentage of cases that met the audit criteria. The performance target was 100%.

This report follows the guidelines for planning, implementing and reporting good quality clinical audits developed by the Healthcare Quality Improvement Partnership.^20^,^21^ Each centre received an individual audit report summarising the main findings, areas of good practice and areas for improvement.

### Ethical considerations

Ethical approval was obtained if required by local legislation. Use of routinely collected data was authorised by participating institutions. Patient confidentiality was ensured by removing identifiable personal data. Individual participant consent was not sought.

## RESULTS

Table 1 and Supplementary Tables S5 and S6 provide a description of the centres selected. Of the 122 TB cases audited, 33 had drug-susceptible and 89 drug-resistant TB (Table 2). The targeted sample of 40 medical records per centre was not reached due to restricted access to medical records (one centre) and fewer TB cases than expected (three centres).

**Table 1:** Characteristics of the five reference centres

Characteristics	Centre 1 (Regional)	Centre 2 (Regional)	Centre 3 (Regional)	Centre 4 (National)	Centre 5 (National)
Recording and reporting system					
Electronic clinical records for TB patients	No	Yes	Yes	Yes	Yes
Electronic laboratory registers	?	Yes	Yes	?	?
Electronic register of contact investigations	No	Yes	Yes	No	Yes
Laboratory services					
Culture methods:					
Solid medium (Löwenstein-Jensen)	Yes	Yes	Yes	Yes	Yes
Liquid, semi-automated system	Yes	Yes	Yes	No	Yes
NAAT for species identification	Yes	Yes	Yes	No*	Yes
DST methods					
Proportion method	Yes	Yes	Yes	Yes	Yes
Liquid medium, semi-automated system	Yes	Yes	Yes	No	Yes
Solid medium, colorimetric method	No	No	No	Yes^†^	No
Line-probe assay	Yes	Yes	Yes	No	Yes
Cartridge-based semi-automated NAAT	Yes	Yes	Yes	No*	Yes
Availbility of anti-TB drugs					
Funding source for drug procurement	Government	Government	Government	Government (FLD) and Global Fund (SLD)	Government
Drug procurement procedure	Decentralised	Decentralised	Centralised (through TB Consilium)	Centralised (through Global Drug Facility)	Centralised (through TB Consilium)
Availability of:					
FLD	Yes	Yes	Yes	Yes	Yes
SLD	Yes	Yes	Yes	Yes	Yes
Fixed-drug combinations	No	Yes	No	Yes	Yes
Bedaquiline	Yes	Yes	No	Yes	Yes
Delamanid	Yes	Yes	Yes	Yes	Yes
Stock-outs reported during 2016–2017	No	No	No	No	No
Infection control measures					
Managerial measures					
Institutional infection control policy	Yes	Yes	Yes	Yes	Yes
Infection control committee	Yes	Yes	Yes	Yes	Yes
Administrative measures					
Triage^‡^	Yes	Yes	Yes	Yes	Yes
Education and training of staff	Yes	Yes	Yes	Yes	Yes
Education of patients	Yes	Yes	Yes	Yes	Yes
LTBI testing and treatment (for staff)	Yes	Yes	Yes	Yes	No^§^
Environmental controls					
Negative pressure ventilation system	Yes	Yes	Yes	Yes	No
Measurement of air changes per hour (frequency)	Yes (annual)	Yes (constant)^¶^	Yes (constant)^¶^	Yes (biannual)	NA^#^
Personal protection					
Protective equipment available	Yes	No**	Yes	Yes	Yes
Surgical mask for patients	Yes	Yes	Yes	Yes	Yes
Particulate respirators for staff	Yes	Yes	Yes	Yes	Yes
Particulate respirators for visitors	Yes	Yes	Yes	Yes	Yes
Respirator fit testing for staff^††^					
Social protection measures					
Access to healthcare services					
TB diagnosis provided free of charge	Yes	Yes	Yes	Yes	Yes
TB treatment provided free of charge	Yes	Yes	Yes	Yes	Yes^‡‡^
Incentives and enablers					
Monthly financial support	Yes	Yes	Yes	Yes	Yes
Food vouchers	—	Yes	Yes	Yes	Yes
Transport costs	—	Yes	Yes	—	Yes
Housing	—	Yes	—		—
Social support through:					
Hospital-based multidisciplinary teams	—	Yes	Yes	—	—
Linkage to care with local health services	Yes	Yes	Yes	Yes	Yes
Integrated community-based case management	—	Yes	Yes	—	—

* Centre 4 introduced cartridge-based NAAT for species identification and DST after 2015.

^†^ The nitrate reductase assay (also known as Kalfin method) was the DST colorimetric method used in Centre 4.

^‡^ Defined as prompt identification and separation of people with TB symptoms.

^§^ In Centre 5, LTBI testing was not done routinely because all staff had been vaccinated using bacille Calmette-Guérin at birth.

^¶^ A centralised, automated and continuous monitoring system was used in Centres 2 and 3.

^#^ Local exhaust ventilation without high-efficiency particulate air filtration was installed at Centre 5 in the sputum induction rooms. Ultraviolet germicidal irradiation is available in waiting rooms and corridors.

** In Centre 2, patients with presumptive (smear-positive) TB were isolated in clinic rooms and asked to wear a mask. Patients did not wear surgical masks at the Outpatient Department to avoid stigma.

^††^ Respirator fit testing was conducted either when the staff started working in the Centre (Centres 1,4 and 5), annually (Centres 2 and 5) or when changing the brand of respirators (Centre 1). The frequency of respirator fit testing in Centre 3 was unknown.

^‡‡^ Patients with multidrug-resistant TB in Centre 5 would seldom need to buy alternative reserve TB drugs, such as thiacetazone, which are neither registered nor provided by the state.

NAAT = nucleic acid amplification test; DST = drug susceptibility testing; FLD = first-line drug; SLD = second-line drug; LTBI = latent TB infection; NA = not applicable.

**Table 2:** Characteristics of TB cases audited in five reference centres

Characteristics	Centre 1 (28 DR-TB) (*n* = 38) *n* (%)	Centre 2 (9 DR-TB) (*n* = 12) *n* (%)	Centre 3 (11 DR-TB) (*n* = 15) *n* (%)	Centre 4 (11 DR-TB) (*n* = 17) *n* (%)	Centre 5 (30 DR-TB) (*n* = 40) *n* (%)
Case definition					
Susceptible	10 (26)	3 (25)	4 (27)	6 (35)	10 (25)
MDR-TB	18 (47)	3 (25)	9 (60)	10 (59)	9 (22.5)
Pre-XDR-TB	9 (24)	4 (33)	2 (13)	1 (6)	13 (32.5)
XDR-TB	1(3)	2 (17)	0	0	8 (20)
Duration of symptoms until diagnosis, days, median [IQR]	90 [45–120]	49 [39–90]	50 [28–139] 180	[60–422]	17.5 [0–30]
Pulmonary TB	38 (100)	9 (75)	13 (87)	17 (100)	40 (100)
Extrapulmonary TB	0	3 (25)	2(13)	0	0
BCG-vaccinated	11 (69)*	2 (33)*	Unknown	17 (100)	1^†^
Previous result of TST recorded	0	0	1 (7)	0	1 (3)
Previous result of IGRA recorded	0	0	1 (7)	0	0
Previous chest radiography result recorded	3 (8)	0	1 (7)	10 (59)	12 (30)
Previous diagnosis of TB	10 (26)	2 (17)	5 (33)	11 (65)	13 (33)
Number of previous anti-TB treatment >1 month, median [IQR]	2 [1–2]	NA^‡^	1 [1–2.5]	2 [1–3]	1 [1–1]
Number of months between last treatment >1 and current diagnosis, mean ±SD	38.8 ±24	NA^‡^	207 ±65.3 16.8	±28.3	88.6 ±70.5
Previous pulmonary surgery	1 (3)	0	0	0	0
Age of patients surveyed in the reference centres, years, mean ±SD	38.0 ±13.4	35.1 ±14.6	45.8 ±14.9 47.6	±12.6	41.1 ±11.7
Males	28 (74)	7 (58)	13 (87)	11 (65)	29 (73)
Foreign-born	32 (84)	12 (100)	3 (20)	0	4 (10)
Unemployed	10 (27)^§^	4 (33)	4 (27)	13 (77)	27 (68)
Homeless patients	3 (8)	0	1 (7)	0	7 (18)
Intravenous drug user	2 (5)^§^	0	4 (27)	1 (6)	6 (15)
Smoker					
Current	14 (39)^¶^	2 (18)^¶^	5 (38)^¶^	12 (71)	28 (80)^¶^
Former	0^¶^	1 (11)^#^	1 (8)^¶^	1 (6)	2 (6)^¶^
Alcohol abuser					
Current	3 (8)	3 (27)**	1 (7)	7 (41)	18 (45)
Former	2 (6)^††^	0^#^	1 (7)	4 (24)	2 (9)^††^
Incarcerated	3 (8)	0	3 (20)	0 (0)	11 (29)^‡‡^

* Denominator = total number of TB patients for whom data on BCG vaccination were available (Centre 1 = 16; Centre 2 = 6).

^†^ Data on BCG vaccination available only for one patient.

^‡^ Data available for one patient only. The patient was previously treated once, 8 years (96 months) prior to the current diagnosis.

^§^ Denominator = total number of TB patients for whom data on unemployment and use of intravenous drugs were available (Centre 1 = 37).

^¶^ Denominator = total number of TB patients for whom data on smoking habits were available (Centre 1 = 36; Centre 2 = 11, Centre 3 = 13; Centre 5 = 35).

^#^ In Centre 2, data on former smoking and alcohol abuse were available for only nine patients.

** Denominator = total number of TB patients for whom data on current alcohol abuse were available (Centre 2 = 11).

^††^ Denominator = total number of TB patients for whom data on previous alcohol abuse were available (Centre 1 = 32; Centre 2 = 9, Centre 5 = 22).

^‡‡^ Denominator = total number of TB patients for whom data on incarceration were available (Centre 5 = 38).

DR-TB = drug-resistant TB; MDR-TB = multidrug-resistant TB; XDR-TB = extensively drug-resistant TB; IQR = interquartile range; BCG = bacille Calmette-Guérin; TST = tuberculin skin test; IGRA = interferon-gamma release assay; SD = standard deviation.

Most TB patients were hospitalised. Patients with drug-resistant TB had longer hospitalisation periods, with a median length of stay ranging between 42 and 233 days. In contrast, the median hospitalisation time for drug-susceptible TB patients ranged between 29 and 80 days. Treatment success rates for all TB cases ranged between 75% and 100% (Table 3).

**Table 3:** Treatment outcomes of TB cases audited in five reference centres

	Centre 1 (*n* = 10) *n* (%)	Centre 2 (*n* = 3) *n* (%)	Centre 3 (*n* = 4) *n* (%)	Centre 4 (*n* = 6) *n* (%)	Centre 5 (*n* = 10) *n* (%)
Drug-susceptible TB patients					
Hospital stay	10 (100)	0	1 (25)*	6 (100)	10 (100)
Total time in hospital, days, median [IQR]	29 [27–37]	NA	NA	68 [64–86.0]	80 [45–92]
Time to sputum smear conversion, days, median [IQR]	27 [17–82]	NA^†^	NA*	30 [30–60]	40 [31–63]
Time to culture conversion, days, median [IQR]	60.0 [60.0–95.0]	NA^†^	15.0 [10.0–60.0]	45.0 [30.0–90.0]	60.0 [49.5–84.5]
Final outcome					
Cured	8 (80)	—	2 (50)	6 (100)	9 (90)
Treatment completed	2 (20)	3 (100)	1 (25)	—	—
Treatment success	10 (100)	3 (100)	3 (75)	6 (100)	9 (90)
Treatment failed	—	—	—	—	—
Died	—	—	1 (25)	—	—
Lost to follow-up	—	—	—	—	1 (10)
Transferred out	—	—	—	—	—
	Centre 1 (*n* = 28)	Centre 2 (*n* = 9)	Centre 3 (*n* = 11)	Centre 4 (*n* = 11)	Centre 5 (*n* = 30)
Drug-resistant TB patients (i.e., MDR-TB, pre-XDR-TB	and XDR-TB)				
Hospital stay	28 (100)	7 (78)	9 (82)	11 (100)	29 (97)
Total time in hospital, days, median [IQR]	42 [27–71]	96 [54–146]	96 [52–107]	233 [209–262]	109 [55–356]
Time to sputum smear conversion, days, median [IQR]	42 [28–60]	43 [18–146]	32 [30–91]	60 [60–60]	395 [29–77]
Time to culture conversion, days, median [IQR]	60 [30–90]	75 [41–146]	60 [32–91]	60 [60–60]	57 [36–92]
Final outcome					
Cured	20 (71)	3 (33)	7 (64)	9 (82)	23 (77)
Treatment completed	1 (4)	6 (67)	2 (18)	—	—
Treatment success	21 (75)	9 (100)	9 (82)	9 (82)	23 (77)
Treatment failed	1 (4)	—	—	—	—
Died	—	—	—	—	2 (7)
Lost to follow-up	3 (11)	—	—	2 (18)	5 (17)
Transferred out	3 (11)	—	2 (18)	—	—

* In Centre 3, one drug-susceptible TB patient was hospitalised during 29 days. The patient’s initial smear microscopy was negative and the culture was positive.

^†^ In Centre 2, one of the three drug-susceptible TB cases had extrapulmonary TB. Data on sputum smear and culture conversion were unknown for the other two cases.

IQR = interquartile range; NA = not applicable; MDR-TB = multidrug-resistant TB; XDR-TB = extensively drug-resistant TB.

All centres had pre-defined diagnostic algorithms which included chest radiography, smear microscopy and culture as initial step. The diagnostic algorithms were adequate (Standard 4, Supplementary Table S7). Laboratory tests were performed in quality-assured laboratories. Additional diagnostic imaging (i.e., computed tomography) was routine in Centre 5; all other centres prescribed it occasionally to assess the extent of lung damage. The tuberculin skin test (TST) or interferon-gamma release assays (IGRAs) were not performed within the diagnostic algorithm for active TB, except for Centre 1. Nearly all drug-susceptible (*n* = 7) and few MDR-TB patients (*n* = 9) in Centre 1 were tested using IGRA alone or TST and IGRA combined. Nucleic acid amplification tests (NAATs) were routinely implemented in Centres 1 and 5. NAATs were not yet available in Centre 4 during the study period, causing a diagnostic delay of 10–14 days in case of semi-automated liquid culture testing, and 42–45 days in case of solid culture in Löwenstein-Jensen medium. The diagnostic algorithm included sequential drug susceptibility testing (DST) at all centres, i.e., DST for second-line drugs was performed only for strains with confirmed resistance to first-line drugs. Most patients with drug-resistant TB were poly-resistant, i.e., resistant to a mean of 5 (range 2–13) TB drugs. Of those with available second-line DST results (*n* = 84), 24% were resistant to fluoroquinolones and 37% to second-line injectable drugs.

Treatment was prescribed with the correct regimen, dosages and duration, regardless of risk factors and DST pattern (Standards 8 and 12, Supplementary Table S8). Fixed-dose combinations for first-line drugs were used at two centres and bedaquiline had been introduced in four centres.

Most patients received at least one type of financial, psychological or social support to facilitate their adherence to treatment (Standard 9, Supplementary Table S8). Treatment monitoring through monthly follow-ups using both sputum smear microscopy and culture (Standard 10) was not implemented universally. Centres 1 and 2 in particular did not follow this standard. Aiming at early detection of relapse, some centres followed up (once or twice a year) MDR-TB patients during a total period of 2–5 years after treatment completion.

Management of adverse events was adequate (Standard 12). Most patients on second-line treatment (85%) had adverse events requiring dose adjustment or replacement with alternative drugs. Therapeutic drug monitoring/pharmacokinetics was done to guide dosing of injectable drugs (eight MDRTB patients in Centre 2; one MDR-TB patient in Centre 3) and antiretroviral treatment (ART) (one patient with MDR-TB-HIV co-infection in Centre 3).

Management of TB and HIV co-infection (Standards 14 and 15) was well implemented, with all patients being offered HIV testing and put on ART when needed in four centres, and almost all in Centre 5 (Supplementary Table S9). All centres reported well-coordinated TB-HIV collaborative activities during hospitalisation. Almost all centres offered integrated ambulatory care for patients with TB-HIV co-infection (Centres 1–4). Comorbidities other than HIV (Standard 17) were also routinely assessed, except for hepatitis in Centre 5.

An electronic TB register was maintained in three centres, providing case-based data for monitoring and evaluation, and allowing for reporting treatment outcomes (Standard 21). Medical records had complete information on prescribed medications, bacteriological response and adverse reactions for all patients (Standard 13). However, laboratory results were not always easily accessible in each patient’s file. All patients had a correct treatment outcome documented in their files (Supplementary Table S9).

Contact investigation was performed for close contacts of all TB patients included in the audit (Standard 18, Supplementary Table S10). Infection control plans/policies with managerial (e.g., infection control committees and planning), administrative (e.g., latent TB infection [LTBI] screening among staff), environmental (i.e., methods used to decrease quantity of droplet nuclei and to control their direction in the air), and personal protection measures (e.g., availability of respirators) were available at all centres (Standard 20, Table 1 and Supplementary Table S10), although coverage/implementation varied (Supplementary Tables S5 and S6). At Centre 4, healthcare staff received training in infection control, and if necessary, additional training on guidelines for TB detection to decrease diagnostic delay among symptomatic respiratory patients. Screening for LTBI among healthcare staff, with either TST or IGRA, was routinely done at four centres for occupational health reasons.

## DISCUSSION

This survey aimed at assessing TB management in different EU/EEA settings against international standards for TB care that were relevant to the study period. Our results showed good adherence to the ESTC in selected TB reference centres. TB case management, with special focus on MDR/XDR-TB, was conducted to a very large extent according to standards for TB care. Similarly, infection control measures were largely implemented according to national and international guidelines.

Several good practices were reported. TB diagnosis was performed using quality-assured and up-to-date laboratory tests. Treatment regimens were adequate and were based on correct dosages. Introduction of bedaquiline enabled treatment of pre-XDR-TB and MDR/XDR-TB patients, in line with international recommendations.^22^–^24^ Other practices such as record keeping, can be further improved. Although case-based records provided detailed accounts of the clinical history, these did not always include notes on social and financial support. Similarly, although patient-centred care provided, its implementation varied according to local arrangements.

A similar audit conducted in 2009–2010 identified several problem areas: 1) surveillance (i.e., missing information on final outcome); 2) infection control (i.e., deficient implementation of administrative and environmental measures); 3) clinical management of TB (i.e., inadequate diagnosis and treatment procedures); 4) clinical management of HIV (i.e., suboptimal HIV counselling and testing, and inadequate ART treatment); 5) laboratory support (i.e., suboptimal/not quality-assured laboratory practices); 6) diagnostic and treatment algorithms (i.e., limited implementation of rapid diagnostic tools); 7) guidelines (i.e., lack of updated, evidence-based guidelines); 8) drugs (i.e., limited availability of all first-and second-line drugs); and 9) funding (i.e., insufficient resource availability for TB prevention and control activities).^3^

Our results show a considerable improvement in TB prevention, care and infection control when compared to the previous audit. We acknowledge that not all previously recruited reference centres were included in our study. However, countries with a similar geographic location and TB incidence were enrolled to obtain comparable results. Few areas still require attention. For example, different algorithms for diagnostic testing were used. NAATs for initial diagnosis and DST were not routinely available at all centres, resulting in a prolonged wait time for results. Timely and universal access to NAATs depends not only on the availability of tests, but also on adequate infrastructure, use of standard operating procedures, and sufficient human and financial resources to ensure sustainability.^17^,^25^

Large differences in hospitalisation periods were also observed. Although TB treatment was initiated during hospital admission in all centres, patients with drug-susceptible and -resistant TB in Centres 4 and 5 remained hospitalised for longer periods. Reduction of hospitalisation length and implementation of community-based models for ambulatory care are cost-effective approaches for the provision of MDRTB treatment.^26^,^27^ Development of context-specific criteria for hospital admission and discharge, and ambulatory management of MDR-TB patients have been suggested for EU countries to minimise transmission and contribute to workplace safety.^25^

Few patients were lost to follow-up or transferred out. The majority of those lost to follow-up were reported from Centre 5. This centre also reported limited patient-centred actions to support treatment adherence. The division between medical and social approaches for the delivery of TB care has previously been highlighted as an important issue.^28^ Fragmentation of support indicates neglect of the social and structural vulnerabilities of TB patients and contributes to loss to follow-up.^28^ Conversely, provision of psychosocial support during treatment improves treatment adherence and retention in care among MDR-TB patients.^29^ A blueprint promoting the uptake and scale-up of people-centred models of care has been developed to inform policy-makers and relevant stakeholders.^30^

As part of this people-centred model of care, integrated treatment of TB and comorbidities needs to be improved. In our study, TB patients were offered HIV testing and counselling as recommended in international guidelines.^6^ However, patients with TB-HIV co-infection were not always offered ART and had to visit separate TB and HIV clinics after hospital discharge. Due to the overlap of risk factors in populations at-risk for TB and hepatitis B and C, it is also likely that TB patients co-infected with hepatitis B and C virus were undiagnosed.^31^

The use of well-known standards of care (i.e., ESTC) and the inclusion of senior consultants with many years of experience in treating MDR-TB patients in the data collection teams contributed to the reliability of our findings. However, we were not able to assess all aspects of TB care in this audit. For example, while information on support provided to foster adherence to treatment was collected, there was no assessment as to whether all patients who needed support received it.

A limitation of our study is the potential sampling bias, introduced by the selection strategy of the TB reference centres. However, different at-risk populations were captured by varying the study settings, such as migrants, including EU and non-EU citizens among those foreign-born; people who inject drugs; and homeless people. Some of the selected centres were regional TB reference centres. Results obtained from regional centres may not be generalisable to national level. Although the audit results were shared with local collaborators, the planning and implementation of a quality improvement action plan were outside the scope of the present study.

In conclusion, the centres included in this study reported good adherence to the standards for TB care recommended for the EU/EEA. Accessible and integrated services for screening and treatment of LTBI and active TB that are responsive to the social vulnerabilities and comorbidities affecting TB patients are needed to reach TB elimination in the EU/EEA.
